# The Matrix Reloaded—The Role of the Extracellular Matrix in Cancer

**DOI:** 10.3390/cancers15072057

**Published:** 2023-03-30

**Authors:** Hans Raskov, Shruti Gaggar, Asma Tajik, Adile Orhan, Ismail Gögenur

**Affiliations:** 1 Center for Surgical Science, Zealand University Hospital, Lykkebækvej 1, 4600 Køge, Denmark; 2 Department of Biomedical Sciences, University of Copenhagen, 2200 Copenhagen, Denmark; 3 Department of Clinical Oncology, Zealand University Hospital, 4000 Roskilde, Denmark; 4 Department of Clinical Medicine, University of Copenhagen, 2200 Copenhagen, Denmark

**Keywords:** extracellular matrix, composition, cancer, desmoplasia, therapeutical targets

## Abstract

**Simple Summary:**

During the development of solid cancers, the ECM undergoes significant changes in composition and function that facilitate the growth and spread of cancer cells. To enhance our comprehension of the disease and integrate ECM profiling into personalized treatment approaches, it is crucial to investigate the biochemical and biophysical characteristics during all stages of carcinogenesis. This review highlights the latest findings in ECM research and potential targets for treatment.

**Abstract:**

As the core component of all organs, the extracellular matrix (ECM) is an interlocking macromolecular meshwork of proteins, glycoproteins, and proteoglycans that provides mechanical support to cells and tissues. In cancer, the ECM can be remodelled in response to environmental cues, and it controls a plethora of cellular functions, including metabolism, cell polarity, migration, and proliferation, to sustain and support oncogenesis. The biophysical and biochemical properties of the ECM, such as its structural arrangement and being a reservoir for bioactive molecules, control several intra- and intercellular signalling pathways and induce cytoskeletal changes that alter cell shapes, behaviour, and viability. Desmoplasia is a major component of solid tumours. The abnormal deposition and composition of the tumour matrix lead to biochemical and biomechanical alterations that determine disease development and resistance to treatment. This review summarises the complex roles of ECM in cancer and highlights the possible therapeutic targets and how to potentially remodel the dysregulated ECM in the future. Furthering our understanding of the ECM in cancer is important as the modification of the ECM will probably become an important tool in the characterisation of individual tumours and personalised treatment options.

## 1. Introduction

**Figure 1:**
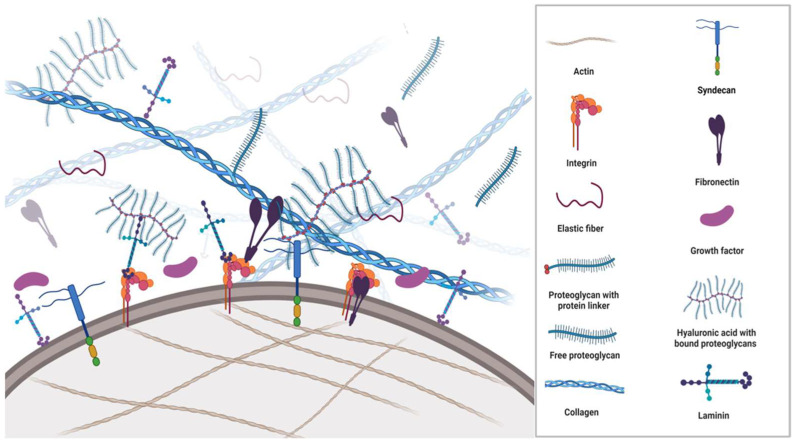
Schematic overview of the ECM structure and its components. The ECM is a complex environment and highly organised support network comprised of multiple proteins such as collagens, fibronectin, proteoglycans, integrins, growth factors and metalloproteinases, which provide cell anchorage. The ECM regulates a plethora of functions to maintain homeostasis. Each matrix protein consists of specific properties that define structural, mechanical, and chemical characteristics. In the figure, a fibronectin molecule is linked to a transmembranous integrin dimer, which is attached to a collagen molecule and thus creates a connection between the cytoskeleton and the ECM. Moreover, laminin complexes are attached to integrins, glycoproteins, and glycolipids through the linker/anchor region (LG domain) on the membrane, creating a dynamic link between cells and the ECM. Syndecans associate with integrins, growth factor receptors, as well as other ECM glycoproteins and collagens. The extracellular domains of syndecans are important for cell–cell and cell–matrix interactions via the glycosaminoglycan sidechains.

All cells synthesise, secrete, and degrade the extracellular matrix (ECM) occupying the space between them. Apart from being passive mechanical support for cells, the ECM is an extraordinarily complex and highly dynamic macromolecular meshwork of proteins, glycoproteins, proteoglycans, water, minerals, and a multitude of bioactive molecules that determine the phenotypes and molecular functions of the cells it surrounds (Figure 1). The interaction between ECM components, ECM-bound factors and cell surface receptors plays a crucial role in mediating cell adhesion and signalling that regulates multiple biological processes. Additionally, the ECM caters to the three-dimensional architectural structures of organs.

Serving as a reservoir for growth factors (GF) and other morphogenic proteins such as matrix metalloproteinases (MMP) originating from tumour cells and non-malignant stromal cells in the microenvironment, the ECM provides positional information to cells and facilitates cell proliferation, differentiation, signalling, and migration. The bidirectional flow of information between cells and the ECM regulates changes in ECM morphology and composition that involves the recruitment and recycling of large numbers of bioactive molecules (reviewed in [1])

The structural scaffolding, mechanical strength, and organisation of ECM components maintain the viability and motility of cells through cell surface contact points that connect the ECM to the cytoskeleton [2,3,4]. The ECM regulates the ability to transport cargo and resist deformation [5,6].

Through cytoskeleton/ECM adhesions and cytoskeleton contractions, cells can sense the spatial context and mechanical properties of the surrounding ECM. Mechanical stimuli convey downstream signalling (mechano-transduction) that regulates gene transcription [7] and continuously remodel the deposition, degradation, and modification of ECM components. The basement membrane (BM) is part of the ECM that separates epithelial cells from the deeper layers of connective tissues where bioactive molecules moving in and out of cells get filtered. The BM is a pericellular matrix structure that functions as a physical barrier and maintains cellular phenotypes without conferring any structural stability to the cell.

In cancer, tumour cells actively remodel their surrounding ECM through a variety of mechanisms, including the secretion of ECM-degrading enzymes and the synthesis of ECM proteins. The resulting dysregulation induces a range of biophysical and biochemical changes, including excess matrix deposition, rigidity, and fibrosis of the stroma [8]. These changes affect the three-dimensional spatial topology of the matrix around cells as well as cell fate promoting tumour growth, tumour cell migration, metastasis and resistance to anticancer therapies [9,10]. Understanding the relationship between cell motility and the characteristics of the extracellular matrix (ECM) is of great importance in cancer research, highlighting the necessity of mapping the intricate biological structures of both the ECM and the tumour microenvironment (TME) during the onset and progression of cancer. As we continue to make significant advancements in exploring intrastromal communication, it is possible that we may soon witness the implementation of cancer therapies that target specific stromal alterations. This article reviews the structural changes and dynamics of the ECM, as well as some of the current investigational drugs and repurposed pharmaceuticals that target ECM dysregulation in cancer.

Blood, being a fluid tissue with a liquid ECM (plasma), is not the focus of this review.

## 2. Components of ECM

ECM is a composite of cell-secreted macromolecules that include fibrous proteins providing the tissues tensile strength and glycoproteins and proteoglycans providing resistance to compression and deformation. Importantly, these molecules participate in multiple signalling pathways and are described in the subsequent section.

## 3. Fibrous ECM Proteins

### 3.1. Collagen

Collagen is a polypeptide structure produced by fibroblasts. Except for the brain, collagen is the most abundant protein throughout the human body and the most significant protein in the ECM [11]. Collagen is the major component of most connective tissues supporting and contributing to the three-dimensional from of organs. In addition, collagen plays an important role in various physiologic processes that include angiogenesis, haemostasis, and mineralisation, as well as in common pathologies such as cancer, fibrosis, and cardiovascular diseases [12].

In solid cancers, collagen deposition not only creates a barrier for cytotoxic immune cells and increases therapy resistance but also provides a rich source of exploitable metabolic fuels for cancer cells [13].

Following synthesis and assembly in the endoplasmic reticulum, the precursor peptide procollagen is packaged and exocytosed into the extracellular space. In the extracellular space, propeptide domains at the carboxy- and amino terminals of the procollagen are cleaved off by MMPs to modify the fibril shape and prevent lateral growth. The formation of the mature collagen microfibril requires binding to the N-terminus of fibronectin [14].

Each collagen fibre is made up of several subtypes. Defined by their bonds and amino acid repeats, twenty-eight different types of collagen composed of at least 46 distinct α-chains have been identified in humans [15]. Nearly 50% of amino acids incorporated into collagens are proline and glycine, which have important roles in the regulation of energy production, protein synthesis, redox balance, and intracellular signalling [16].

Collagen can be divided into fibrillar collagens type 1, 2, 3, 5, 11, 24, and 27 and non-fibrillar collagen type 4 (basement membrane); 6 (beaded filaments); 7 (anchor fibres); 8, 10 (short chain); 9, 12, and 14 (fibril-associated collagens with interrupted helices or FACIT); and type 13 (transmembrane collagen). The most abundant is collagen type 1, found in the skin, bones, and tendons [17].

Mutations in collagens 1, 2, 3, 9, 10, and 11 result in a broad range of ailments affecting cartilage, bones, and blood vessels, including osteogenesis imperfecta, various types of chondrodysplasia, Ehlers–Danlos syndrome types 4 and 7, and some cases of osteoarthritis, osteoporosis, and familial aneurysms [18].

### 3.2. Elastin

Elastin is a fibrillar hydrophobic matrix protein that, in contrast to collagen, is able to stretch eight times its resting length [19]. Elastin provides flexibility to blood vessels, skin, lungs, and ligaments. It is synthesised by fibroblasts, vascular smooth muscle cells [20], smooth muscle cells, and several types of epithelial cells. Being the primary ECM protein in arteries, where it amounts to ~50% of the weight [21], it has an impressive ability to withstand the mechanical stress of more than 3 billion expansions and contractions during an 80-year life cycle.

The precursor protein, tropoelastin, is secreted with a chaperone molecule that facilitates the correct folding of the protein before it is incorporated into the highly flexible elastin strands. Similar to other ECM proteins, such as collagens, mature elastin is extensively cross-linked with other elastin molecules to form sheets and fibres [22]. Elastic fibres are composed of approximately 90% elastin, whilst the remaining components are primarily comprised of fibrillin glycoproteins. Due to its unique structure, extensive cross-linking and durability, elastin does not undergo significant turnover in healthy tissues where it has a half-life of more than 70 years [23]. It is primarily deposited during prenatal development and childhood and is rarely synthesised during adulthood [24]. Aberrant expression of elastases and degradation of elastin trigger the release of elastokines (fragments of matrix proteins with cytokine-like properties) that promote angiogenesis and regulate cell adhesion, chemotaxis, migration, and proliferation. Much of the elastokine effects are mediated by membrane elastin receptor complexes [23] that trigger signalling pathways involving the extracellular signal-regulated protein kinases 1 and 2 (ERK1/2) and serine/threonine-protein kinase (AKT) activation [25]. Elastases belong to the enzyme classes of MMP, aspartic proteases, serine proteases, and cysteine proteases. The destruction of elastin promotes the development and progression of different pathological conditions, including chronic obstructive pulmonary disease, atherosclerosis, vascular aneurysms, and cancer. In lung and colon cancer, the degradation of the matrix and fragmentation of elastin was found mainly to occur at the invasive front, and the expression levels of MMP also correlated to the metastatic potential of these cancers [26,27].

## 4. The Glycoproteins

Glycomics is an important new frontier in life science research. Similar to proteoglycans, glycoproteins are composed of proteins with attached saccharide chains; however, the glycoprotein side chains are much shorter than the saccharide chains in proteoglycans. They contain no (or few) repeating units and are usually branched. The two most important ECM glycoproteins are fibronectin and laminin.

Glycoproteins often act as connecting molecules that bind other ECM molecules, GF, and receptors. They have N-linked and O-linked saccharide sidechains, with the N-linked chains being connected to -NH_2_ on asparagine residues in the protein and O-linked chains to the -OH on the serine/threonine residues. N-linked and O-linked glycoproteins are mainly located on the cell membrane, where they play crucial roles in the cell–cell communication, adhesion, migration, proliferation and healing processes; they may also exist as secreted proteins.

### 4.1. Fibronectin

Within the body, fibronectin exists as soluble plasma glycoproteins (synthesised by hepatocytes and secreted into the blood) and as insoluble cellular fibronectin (a fibrillar cross-linked structure on the cell membranes). It is responsible for cell adhesion, proliferation, migration, and the deposition of ECM proteins [28].

The basic structural unit of fibronectin is a dimer composed of two nearly identical polypeptide chains linked by a pair of disulphide bonds. Fibronectin fibrils serve as mechanical links between the cytoskeleton and the surrounding ECM. It primarily binds to actin-anchored integrins on the cell membrane (Figure 1). Mediating the adhesion of BM components to ECM structures, integrins are heterodimeric (α and β subunits) cell-surface receptors and bi-directional transducers of biochemical signals and mechanical forces acting on the ECM. The α and β subunits both have a cytoplasmic tail, a transmembrane domain, and a large extracellular domain that bind numerous ECM ligands. The anchorage to the ECM is required for normal cells to enter the S phase, even in the presence of GF. If cells detach from their integrin ligation points and lose the sense of their mechanical environment, they undergo a specific type of apoptosis, anoikis (Greek for homeless). Resistance to anoikis is a characteristic feature of tumour cells that enables them to survive under non-adherent conditions [29,30,31].

The connection between the ECM and cytoskeleton stimulates cell proliferation and angiogenesis through pathways that include ERK 1/2 phosphorylation, dysregulation of the HIPPO (tumour suppressor) pathway, and suppression of apoptosis through the nuclear factor kappa B (NF-κB) or the phosphoinositide 3-kinase (PI3kinase)/AKT pathway [32].

Fibronectin fibrillogenesis is initiated by cytoskeleton-derived tensional forces transmitted across transmembrane integrins, typically α5β1 [33]. During this process, soluble molecular fibronectin is irreversibly assembled into insoluble fibrils that stretch up to four times their resting length, which implies domain unfolding and subsequent ECM remodelling [34]. Fibronectin fibres are proposed to be held together by hydrogen and disulphide bonds; however, catalytic agents such as thermolysin, plasmin, thrombin, trypsin, cathepsin D, and chymotrypsin can cleave them.

Fibronectin fibrillogenesis and collagen fibrillogenesis have a complex relationship, with fibronectin regulating the assembly of collagen and vice versa [35]. How the production, organisation and matrix deposition of fibronectin are regulated by tumour cells is less understood as the turnover of fibronectin is largely unexplored [36].

Interacting with other ECM proteins, including GF, glycosaminoglycans, cell surface receptors and other fibronectin structures, fibronectin provides key mechanical and chemical signals to induce differentiation and epithelial-mesenchymal transition (EMT) [37].

Transforming growth factor β (TGFβ), fibroblast growth factor (FGR), platelet-derived growth factor (PDGF), hepatocyte growth factor (HGF), and vascular endothelial growth factor (VEGF) have multiple binding sites within fibronectin. The binding of TGFβ1 to fibronectin fibrils was shown to upregulate EMT [37], whereas dysregulation of fibronectin promoted tumorigenesis and fibrosis, with the expression levels of fibronectin being significant prognostic factors in several cancers [38,39].

Hypoxia-induced factors upregulated in tumour cells stimulate endogenous FN synthesis. Intercellular signalling between tumour cells and protumorigenic stromal cells, such as tumour-associated macrophages, cancer-associated fibroblasts, and myeloid-derived suppressor cells drive persistent FN deposition and remodelling of the ECM that facilitate growth and dissemination [40,41,42].

### 4.2. Laminin

Laminins are one of the major glycoproteins in the basement membranes that glue cells and tissues together and regulate cellular activities and signalling pathways. Structurally, laminins are cross-shaped, trimeric glycoproteins of 400–800 kDa in size and composed of a few distinct domains, of which 16 different combinations have been identified. Primarily involved in tissue repair and wound healing [43,44,45], all laminin complexes have a high affinity for GF through their heparin-binding domains; thus, apart from contributing to the anchoring of cells, laminin is a storage facility for GF whose release determines cell differentiation, survival, shape, and motility [46]. In hepatocellular carcinoma, laminin was found to be involved in EMT and disease progression [47]. The association between ECM proteins and GF is shown in Table 1.

## 5. Proteoglycans

The proteoglycans (mucoproteins) are composed of a protein core covalently attached to glucosaminoglycans (mucopolysaccharides), such as chondroitin sulphate, heparan sulphate or keratan sulphate (Figure 1). Proteoglycans have excellent water retention, gel-forming and space-filling functions [48], conveying resistance to compression and deformation to cells. Although one of the least abundant components in the ECM, they are integral in maintaining a healthy ECM.

Syndecans are transmembrane proteoglycans with heparan and chondroitin sulphate chains attached to their extracellular domain. They may also exist as soluble extracellular domains. Similar to many proteoglycans, they interact with a multitude of ligands, such as GF, adhesion receptors, proteinases, cytokines, chemokines and other ECM proteins to initiate downstream signalling responsible for proliferation, adhesion, angiogenesis, and inflammation [49]. Elevated levels of syndecan expressions in cancer can be correlated with poor outcomes, e.g., of Syndecan-1 in breast cancer and of Syndecan-2 in colorectal cancer, where it is highly associated with metastasis [50].

### Hyaluronic Acid

Hyaluronic acid (HA or hyaluronan) is a hydrophilic glycosaminoglycan. HA synthases in the cell membrane mediate the alternate addition of glucuronic acid and N-acetylglucosamine in a growing chain of thousands of disaccharides [51] that are translocated out of the cell during biosynthesis. HA is abundantly present in the ECM of weight-bearing joints and the interstitial gel [52,53].

**Figure 2:**
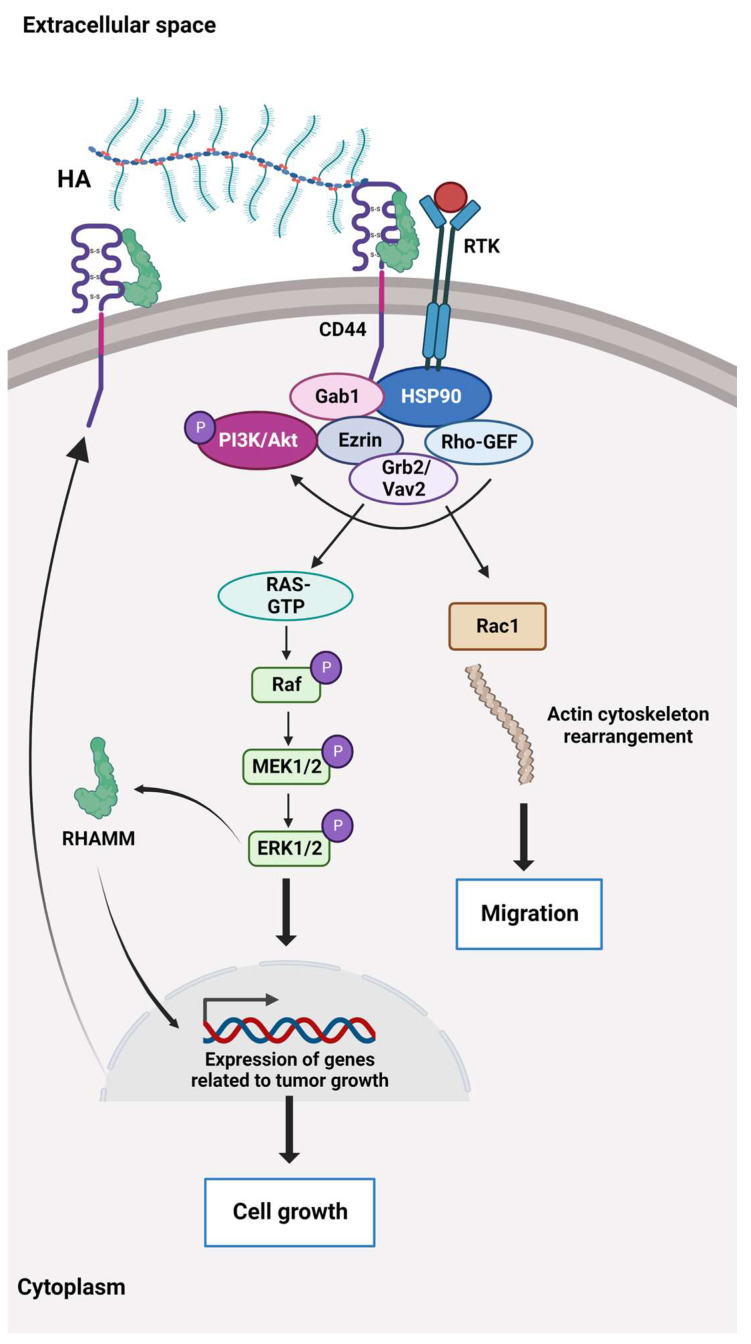
The HA-dependent CD44 signalling. CD44 and the receptor for hyaluronic acid (HA)-mediated motility (RHAMM) are the main HA receptors. They are commonly overexpressed in cancer, where they activate signalling pathways related to disease progression. CD44 also interacts with other ligands, such as collagens and matrix metalloproteinases, and as such, a multifunctional receptor mainly involved in proliferation, differentiation, migration, and angiogenesis. In contrast to membrane-bound receptors containing signalling domains, RHAMM (CD168) does not contain signal sequences. RHAMM is localised inside the cell and is exported to the cell surface in response to stimuli such as cytokines, including TGF-β. Extracellularly, RHAMM associates with CD44, and when HA binds to these cell surface receptors, it triggers several signalling pathways and complex formation between CD44 and its co-receptors and increases the expression of TGF-β receptors. Activation of downstream effectors, e.g., Akt, PI3K, MEK1/2, ERK1/2, and Ras/Raf/Rac, results in the expression of a variety of inflammatory cytokines and activation of a feedback loop augmenting cell surface expression of CD44/RHAMM. The CD44/RHAMM complex stimulates cell motility and increases angiogenesis by promoting the migration of endothelial cells towards the tumour. Further, these signalling events drive proliferation, invasion, and cytoskeletal rearrangements, leading to normal cell functions, such as fibroblast migration and immune cell function, and tumour growth and progression. ERK1/2: Extracellular signal-regulated protein kinases 1 and 2, Ezrin: kinase substrate protein, Gab1: PAR-1 kinase, Grb2/Vav2: growth factor receptor-bound protein2/guanine nucleotide exchange factor, GTP: Guanosine triphosphate, HSP90: Heat shock protein 90, MEK1/2: Mitogen-activated protein kinases 1 and 2, PI3K/Akt: Phosphoinositide 3 kinase/protein kinase B (originally Ak strain transforming kinase), Ras/Raf/Rac: Rat sarcoma virus protein/rapidly accelerated fibrosarcoma protein kinase/Ras-related C3 botulinum toxin substrate 1, Rho-GEF: Rho-guanine nucleotide exchange factors, RTK: Receptor tyrosine kinase.

HA binds to the cluster of differentiation 44 protein (CD44), a transmembrane receptor that participates in many physiological and pathological processes by interacting and activating key signalling cascades (Figure 2). Ligation of CD44 initiates the expression of genes related to tumour growth, proliferation, and survival, and its ligation with HA induces cytoskeletal rearrangements and membrane ruffling that leads to active cell migration [54,55]. Further, CD44 serves as a marker for several types of stem cells [56,57].

HA turnover occurs in various molecular weights of HA distinguished by their polymer length, e.g., high-molecular-weight HA (Mw > 1.8 × 10^6^ Dalton) and low-molecular-weight HA, Mw 4–10 × 10^5^ Dalton), The molecular weight of HA determines its activities. High-molecular weight HA inhibits mitogenic processes and possesses anti-inflammatory effects, whereas low-molecular weight-HA shows protumorigenic effects by enhancing proliferation and inflammation [58].

Within a solid tumour, HA is deposited by cancer-associated fibroblasts (CAF) and cancer cells and is a major structural component of the TME [59].

HA plays a central role in cancer cell proliferation and migration. HA recruits tumour-associated fibroblasts, macrophages, and HA fragments to promote angiogenesis and immunosuppression, either directly or through macrophage protumorigenic polarisation [60,61]. Moreover, increased HA levels in the TME are associated with poor prognosis and survival in several cancers [54,62]. The recruitment and polarisation of macrophages may be used in future targeted anticancer therapies. Chimeric antigen receptors (CARs) have become a promising approach to increasing tumour cell recognition by cytotoxic immune cells. CAR-T cells are already in clinical use. In vitro, tumour-associated macrophages engineered with CAR constructs can be directed at tumour antigens and kill tumour cells by phagocytosis. Furthermore, the CAR-HER2-CD147 construct activates the expression of matrix metalloproteinases, degrades the ECM, and overcomes the physical barrier that prevents the infiltration of cytotoxic immune cells. As the degradation of the ECM may also support the dissemination of tumour cells, any treatment involving metalloproteinases must be calibrated meticulously [63].

Second-generation CAR-M cells are in development. In addition to maintaining the characteristics of first-generation CAR-M technology, the goals of second-generation therapies comprise improving tumour-associated antigen presentation and T-cell activation [64].

## 6. Sensing and Communication in ECM

Mechanotransduction allows living organisms to receive and respond to mechanical forces from the internal and external environment. Mechanically activated ion channels represent the primary mechanism for mechanotransduction that effectively converts mechanical stimuli into electrochemical signals. In the cell membrane, the transmembrane and mechanotransducing Piezo proteins (1 and 2) form ion channels that are activated by pressure. When pressure is applied to the cell membrane, the large, three-bladed propeller-shaped molecular complexes flatten and stretch, whereby the ion channels open and allow a flow of calcium and other positively charged ions through the central pore module into the cell, whereby biochemical signals are created.

Cells probe their environment mechanically via lamellipodia (membrane protrusions composed of a dense and dynamic network of actin filaments). Lamellipodia decode the mechanical feedback and resistance from their surroundings [65] through membrane integrins and syndecans. These molecular complexes trigger intracellular signalling cascades involving the unfolding of proteins associated with the contractile actin cytoskeleton, such as Rho-associated protein kinase [66]. When the actin protein polymerises to form filaments, it enables the cell to control shape and mechanics—as used by crawling and phagocytising immune cells and migratory tumour cells.

In cancer, the cytoskeleton signalling reduces the number of intercellular adhesion molecules, induces EMT, upregulates membrane integrins, and enhances the migratory potential [7] and therapeutic resistance [67]. The mechanotransduction that arises from changes in ECM stiffness shifts cytoskeletal dynamics and releases mechanosensitive molecules such as vinculins, paxicillins, and talins that also regulate adhesion and migration [68,69]. Mechanosensitive cytoplasmic proteins connect integrin complexes to the nucleus through linker protein complexes that allow a direct transmission from ECM to the nucleus as reviewed in [70].

The mechanisms leading to gene transcription are not mapped fully. Yes-associated protein 1 (YAP) and WW-domain-containing transcription regulator 1 (TAZ) are transcriptional coactivators that are translocated to the nucleus together with β-catenin upon cytoskeletal tension [71,72,73]. YAP/TAZ activation upregulates the genes associated with proliferation and dedifferentiation, and the nuclear translocation of β-catenin directly destabilises intercellular adhesions, all contributing to EMT for migration through the ECM [74,75]. Simultaneously, the levels of several integrins are upregulated in several tumours. Many signalling pathways are affected by ECM dysregulation, as shown in Table 2.

Integrins activate various intracellular signalling pathways that promote cell survival, growth, and proliferation and targeting these molecules could be an effective strategy to strike at tumour cells. Integrins play key roles in various other diseases, such as ulcerative colitis, cardiovascular diseases and osteoporosis, but they have not been targeted extensively (reviewed in [76]). Kindlin-2 is a widely expressed protein that is critical for integrin-mediated cell–ECM adhesion and signalling. Kindlin-2 localises to the adhesion sites where the ECM molecules are connected to the actin cytoskeleton and increase proline synthesis through interaction with pyrroline-5-carboxylate reductase 1 (PYCR1). PYCR1 is a key mitochondrial enzyme that facilitates the last step in glutamine-to-proline conversion, and its overexpression of PYCR1 is involved in the progression of several cancers, including breast and lung cancer [77]. The increased proline synthesis is linked to increased production and stiffness of the ECM and plays an important role in tumourigenesis [78].

## 7. ECM in Solid Cancers

Tumour progression requires continuous interaction between the ECM and tumour cells where increased cytoskeleton signalling reduces the number of intercellular adhesion molecules, induces mesenchymal transition, upregulates membrane integrins, enhances the migratory potential [7] and therapeutic resistance [67]. Escalated deposition and cross-linking of collagen interfere with cell polarity, cell adhesion, and integrin signalling and promote tumour progression. ECM degradation and loss of BM integrity due to MMP are hallmarks of invasive lesions [79]. Another cancer hallmark is the tumour cell production of glycoproteins and proteoglycans with altered glycosylation (e.g., cancer antigen 19-9 and 125, carcinoembryonic antigen, prostate-specific antigen and alpha-fetoprotein) secreted or shed from the cell membranes into the bloodstream where they may serve as tumour-associated biomarkers) [80].

ECM—the largest part of a solid tumour—applies mechanical and non-mechanical forces on tumour tissue, such as stiffening, increased interstitial fluid pressure, collapsing vessels, hypoxia, and acidity [81] and compromises the outcome of oncological treatments and prognosis [82].

In melanoma and breast cancer, increased amounts of collagen I correlated with disease progression and reduced survival [83,84,85]. Further, collagen and fibronectin are involved in angiogenesis. Interacting with α1β1, α2β1, ανβ3, and ανβ5 integrins, collagen I activates mitogen-activated protein kinase (MAPK) pathways supporting the survival of endothelial cells, remodelling the actin cytoskeleton, and influencing the cells to form lumens [86]. Fibronectin plays a pivotal role in the assembly of vascular matrix components [87], while MMP releases GF, VEGF, and chemokines bound within the ECM [88,89] to remodel the ECM and direct endothelial migration and capillary movements along aligned ECM fibres [90,91].

When nutrient levels run low, integrin-bound glycoproteins of the ECM are easily endocytosed and internalised into lysosomes by tumour cells. The role of ECM as a nutrient provider represents a potential therapeutic target to inhibit integrin uptake in stromal-enriched cancers [92]. Ligand-bound integrin trafficking has recently been shown to affect nutrient signalling through the rapamycin (mTOR) signalling pathway [92,93]. With sufficient nutrient availability, mTOR induces anabolic processes such as protein, nucleotide, and lipid biosynthesis and inhibits lysosomal biogenesis and cellular autophagy [94] through two independent complexes of mTOR. The complex mTORC1 adjusts cell growth and proliferation in response to GF and amino acids, while mTORC2 is involved in actin organisation and cell proliferation and survival [95]. During nutrient starvation, the activity of mTORC1 is downregulated, allowing cells to use other sources of nutrient acquisition, such as autophagy [96,97].

The ECM-attached cells induce adaptive responses or compensatory homeostatic feedback loops, leading to the induction of several pro-survival proteins, including receptor tyrosine kinases and antiapoptotic proteins including the activation of MAPK, PI3K, AKT and the human epidermal growth factor 3 (HER3) [98].

The alignment of collagen fibres by lysyl oxidase (LOX) produced by cancer cells and CAF directs cancer cell migration and induces their proliferation [99,100,101]. In an in vitro study on benign and malignant human ovarian cell lines, the ability to rapidly remodel the matrix enabled tumour cell migration along aligned fibres and changed direction according to microenvironmental cues [102].

The ECM is a storage facility for nutrients, proteases, morphogens, and GF that create pro-migratory gradients. These molecules are released by proteolytic degradation of the ECM, which regulates the rate and intensity of migration [103]. Due to the ongoing modulation of the ECM, the TME is rich in protease-digested fragments that may influence the metastatic potential and apoptosis of cancer cells [104]. These fragments may even display opposite effects as compared to their molecules of origin, making studies on the TME even more challenging [105].

Notably, the binding of an ECM-associated GF to its receptor (GFR) may prevent its endocytosis and secure prolonged, upregulated signalling by the ECM-GF/GFR complex [106]. In addition, cells may generate different responses to the same effector molecule bound to different matrix molecules under the same GF conditions [107].

## 8. Desmoplasia and CAF

Many human solid tumours, such as pancreatic, breast, lung, and colorectal cancers, are characterised by a pronounced stromal reaction where cancer cells and CAF produce vast amounts of matrix molecules required for the growth of the dense, fibrous tissue called desmoplasia [108]. In these tumours, CAF and their collagen matrix products are major components of the stroma and of the tumour mass. The switching of normal fibroblasts into CAF is a fundamental step in the development of a solid tumour and requires epigenetic modifications and tumour-derived exosomal delivery of genetic material [109]. The rewiring of fibroblasts initiates an increased and inexhaustible synthesis of ECM proteins. In a preclinical model of gastric cancer, it was shown that TGFβ and increased SMAD protein activity (driving the expression of alpha-smooth muscle actin/αSMA) provide CAF with a highly contractile phenotype (myofibroblast/αSMA+ fibroblast) that induced tumour cell invasion of the ECM and lymphatic vessels [110].

Nearly all αSMA-expressing fibroblasts are hedgehog-responsive, which makes it a promising therapeutic target [111]. However, in a murine study on pancreatic duct adenocarcinoma (PDAC), the deletion of the hedgehog signalling mediator smoothened from the epithelium had no impact on disease progression [112]. A clinical trial combining chemotherapy with a hedgehog inhibitor failed to show benefits and was terminated due to reduced overall survival [113]. Another clinical trial targeting stromal myofibroblasts in PDAC resulted in a paradoxical accelerated disease progression [114], and thus, antistromal therapies remain controversial.

In general, CAF activation leads to the massive production of cytokines, chemokines, GF, and transcription factors (e.g., PDGF, VEGF, HGF, prostaglandin E2, interleukin (IL)-6, IL-8, tumour necrosis factor, NF-κB, stromal cell-derived factor 1 (SDF1/CXCL12) that regulate the communication with other cells in the TME. Some CAF may even express the major histocompatibility complex and CD74 and activate CD4^+^ T cells in an antigen-specific fashion in a model system, confirming an immune-modulatory capacity [115]. In a PDAC mouse model, the depletion of CAF reduced desmoplasia, but simultaneously, immunosuppression was induced by a reduced number of infiltrating lymphocytes. The tumours became highly undifferentiated, intratumoral blood vessels were diminished, hypoxia and necrosis were evident, and the survival of the animals was shortened [116].

Human PDAC is characterised by extensive desmoplasia and collagen deposition produced by myofibroblasts that are chronically activated by GF secreted by tumour cells. These tumours have high epithelial signal transducer and activator of transcription 3 (STAT3) activity and develop stiff, matricellular-enriched fibrosis associated with high epithelial tension and short patient survival [117]. By mechanosensing, matrix stiffness recruits and activates myofibroblasts from adjacent soft tissues. As these cells produce excess amounts of collagen, the increasing stiffness enhances the process in a positive feedback loop [118].

## 9. ECM Stiffness

ECM stiffening is essential for a tumour to increase in size, displace, and invade neighbouring tissues. Although the interior of a solid, desmoplastic tumour is subject to compression, the hydrated, gel-forming hyaluronan within the tumour conveys tumour cells with resistance to compressive stress [119]. Tumour growth can reorganise collagen fibres and change and stretch the fibre orientation toward the tumour circumference [120]. In a murine model using human head and neck squamous cell carcinoma cells, cellular mechanosignaling induced a progressive linearisation and thickening of collagen that augmented cell growth and survival and created pathways for migratory tumour cells that led to increased metastasis [121].

In squamous cell carcinoma, the stiffness of the TME was shown to have profound effects on the metabolism of protumorigenic stromal cells and cancer cells as both cell populations switched metabolism to increased glycolysis and glutamine consumption [96]. As driver gene mutations direct cancer cells away from oxidative phosphorylation towards anaerobic glycolysis, energy production and biosynthesis accelerate many times while tumour-secreted factors degrade peripheral tissues to fuel disease progression. This metabolic switch provides cancer cells with the ability to survive, thrive, and multiply within a hostile environment characterised by hypoxia, acidity, and low nutrient levels.

In invasive ductal carcinomas, collagen production shifted toward collagen type I and type III compared to benign mammary lesions [122], and in melanoma, increased collagen I expression was correlated with invasiveness, angiogenesis, and reduced survival [123].

## 10. Therapeutical Targets

Processes leading to increased production of matrix molecules, matrix deposition, maturation, and remodelling represent potential treatment targets (Table 2). However, the abundant and highly cross-linked ECM is a barrier to intratumoral drug diffusion, and very few compounds have reached the clinic. Hypoxia, acidity, and deprivation of nourishment in the TME activate antiapoptotic and angiogenic pathways that further increase drug resistance.

Mechanisms of action and ECM targets of investigational new drugs used in recently completed trials are shown in Table 3. Well-known drugs already approved in other indications are being repurposed and investigated in clinical cancer trials. Several of these drugs appear to have an impact on the ECM, e.g., losartan, metformin, tamoxifen, and doxycycline.

Losartan has gained interest as an ECM modulator, and its first use as an antifibrotic agent in cancer was described in 2011, where losartan was shown to inhibit the production of collagen I by CAF isolated from breast cancer biopsies [124]. Losartan (Cozaar) is an angiotensin II receptor 1 blocker that reduces collagen I secretion, inhibits GF release, and suppresses tumour growth in vivo and in vitro [125]. Losartan inhibits the downstream signalling that includes MAPK, JAK2, AKT, NFκB, and HIF-1α [126]. By blocking angiotensin II receptor signalling, losartan prevents angiotensin II from increasing the levels of thrombospondin-1, a major activator of TGF-β, and thus suppresses TGF-β signalling and other profibrotic signals. Preclinical trials with models of breast cancer and PDAC showed that losartan reduced the amount of collagen and hyaluronan intratumorally and significantly improved perfusion by decompressing tumour vessels [127,128]. Seven clinical trials are currently recruiting in PDAC, breast cancer, osteosarcoma, and myelogenous leukaemia (ClinicalTrials.gov, accessed on 1 February 2023).

Metformin suppresses tumour progression by inhibiting the NADH dehydrogenase in the mitochondrial complex I in the electron transport chain and reducing the expression of HIF1-targeted genes, including *VEGFA*. Metformin causes a decrease in hypoxia-inducible factor 1 alpha (HIF-1α) due to destabilisation of the HIF molecules, which is meant to most likely be a consequence of its action on the mitochondrial complex I [129]. In addition, metformin may have an essential antitumour role in the invasion and metastasis pathways by downregulating the expression of MMP-2 and MMP-9 and blocking the activity and the nuclear translocation of NF-κB [130,131]. Clinical trials have reported a reduction in cancer-related mortality in different meta-analyses. However, the results were not significant or univocal [132,133,134].

Tamoxifen is known as an estrogen-receptor antagonist that is most widely used in estrogen-receptor-positive breast cancer and less frequently in cancers of the female reproductive organs. More than 90 trials are currently recruiting in breast cancer, endometrial cancer, lymphomas, and glioblastomas (see ClinicalTrials.gov).

In preclinical trials, and in addition to the antihormone effects, tamoxifen acts as an ECM modulator by regulating the level and activity of collagen cross-linking and ECM degrading enzymes, and hence the organisation of the ECM. It reduces HIF-1A levels by suppressing myosin-dependent cellular contractility and ECM stiffness mechanosensing [135].

Doxycycline is a well-known and well-tolerated antibiotic substance of the family of tetracyclines. It possesses strong MMP inhibitory activity already observed at sub-antimicrobial dosage levels [136]. Doxycycline is recognised to have antitumour properties in various human cancer cell lines, e.g., PDAC [137], colon cancer [138], and melanoma [139].

Doxycycline blocks ECM and membrane degradation by suppressing MMP function. Moreover, doxycycline eliminates the secretion of vascular endothelial growth factor (VEGF) [140].

Clinical trials aim at blocking the oncogenic signalling of GF and Hedgehog proteins as well as the degradation of ECM macromolecules by MMP and cathepsins (Table 3). The ECM degradation by collagenases and hyaluronidases may increase immune cell infiltration and are currently under investigation. Degradation may also release ECM-anchored cytokines and GF leading to progression and dissemination [8].

MMP degrade ECM molecules, and their cleavage products regulate chemotaxis, migration, and angiogenesis as well as tumour cell growth, differentiation, and apoptosis. As MMP promote tumour development by increasing invasiveness and the growth of tumour cells, their levels increase with the cancer stage and can be used as diagnostic and prognostic biomarkers. The lack of specificity of MMPs inhibitors, including Ilomastat, Marimastat and CGS-27023A, has led to dose-limiting adverse events such as arthralgia, myalgia, tendinitis, musculoskeletal syndrome, and gastrointestinal disorders and more than 50 MMP inhibitors have so far proven unsuccessful in clinical studies [141]. However, targeting highly specific antibodies could provide clinical benefits without severe side effects, as reviewed in [40].

Discoidin domain receptors (DDR) are upregulated in many cancers and spontaneously bind to collagen in an unregulated manner. DDR sense ECM stiffness, and they transmit signals into cells unidirectionally. DDR signalling may be blocked by tyrosine kinase inhibitors such as imatinib, which has been used for several years in cancer treatment and bivatuzumab, a CD44 (HA receptor) antibody evaluated in clinical trials.

Strategies targeting another HA receptor, RHAMM, and the angiotensin II type 1 receptor have attracted much attention as moderate efficacy was demonstrated in preclinical and clinical trials [142].

TGFβ signalling is a prominent target to inhibit collagen synthesis. Apart from excessive deposition of collagen, overexpression of TGFβ causes multiple metabolic dysfunctions that promote EMT, immune dysfunction, and fibrosis. The targeting of TGFβ signalling appears to be a promising strategy, and trabedersen (TGFβ2 antisense nucleotide), fresolimumab (TGFβ monoclonal antibody) and galunisertib (TGFβR1) are some of the promising drugs targeting this pathway (Table 2). Combinatory regimens with cytotoxic or immunomodulatory drugs should be studied further [143].

Retinoids have been shown to regulate cell growth, differentiation, and apoptosis [144]. All-trans retinoic acid (ATRA) is used in the treatment of promyelocytic leukaemia. In solid tumours, ATRA induces CAF quiescence, reduces desmoplasia, and potentially enhances the delivery of chemotherapy intratumourally [145]. In a clinical phase I trial on 27 patients with advanced, unresectable PDAC, ATRA proved safe and tolerable [146]. ATRA is now evaluated in a phase II randomised clinical trial in locally advanced PDAC (NCT04241276) and has over 40 ongoing phase 3 trials for several cancer types.

Pirfenidone, an antifibrotic drug approved for lung fibrosis reduces TGFβ activity, CAF activation and collagen I and II depositions. Results are awaited for the phase 1 study investigating its use in advanced solid tumours (NCT04324372).

Pamrevlumab, which inhibits connective tissue GF and decreases matrix deposition, has two ongoing trials in locally advanced, unresectable pancreatic cancer (NCT03941093) and stage 4 pancreatic cancer (NCT04229004).

Nintedanib, an oral triple angiokinase inhibitor of VEGFR, PDGFR, and FGFR known to reduce fibrosis, has already been approved for lung adenocarcinoma and is being heavily investigated in other cancers (Table 2).

Blocking LOX inhibits the formation of cross-linkages and stabilisation of collagen and elastin fibres, thereby reducing tissue stiffness. Aside from the few LOX inhibitors that were clinically ineffective, PXS-5382A targeting LOXL2 (NCT04183517) and PXS-5505, a pan LOX inhibitor, is still under investigation (NCT04676529 and NCT05109052).

In terms of hyaluronidases, PEGPH20 as monotherapy did not show any clinical benefit. A few clinical trials are currently evaluating the therapeutic effects of combining hyaluronidase and chemotherapeutics (Table 2). PEGPH20 showed clinical benefits with chemotherapy in PDAC in a phase 2 trial but did not show any clinical benefit in phase 3 trials [58].

Combinatory regimens of already identified drugs should be studied further. In future, tumour ECM proteins could serve as antigens for cancer vaccines and CAR therapies.

## 11. Discussion

The plasticity and dynamics of the ECM are essential to all stages of cancer progression and are recognised as important targets for intervention. Nonetheless, targeting the ECM is challenging as it may affect normal physiological processes leading to off-target effects.

In solid tumours, the increasing desmoplasia upregulates fibrotic genes that accelerate the deposition of ECM material and sustain a favourable environment for tumour cells. Improving insight into the tight cellular control of ECM molecule trafficking reveals new perspectives in the manipulation of the ECM architecture that may ease the delivery of therapeutics and prevent the migration and dissemination of tumour cells.

In advanced, metastatic stages of PDAC and colorectal cancer, regimens of ECM-degrading compounds such as LOX and HA inhibitors combined with conventional chemotherapeutics have so far failed to show additive or synergistic effects most likely due to the large, impenetrable ECM of late-stage cancers. Therapies interfering with the ECM may have more pronounced effects in the early stages of carcinogenesis before the dissemination of tumour cells. Theoretically, ECM modulation in early carcinogenesis may also impact the establishment of pre-metastatic niches in target organs.

To improve drug penetration and increase the infiltration of immune cells into tumour tissues, biocompatible and non-immunogenic nanoparticle-based drug delivery systems using customised drug release profiles and carrying ECM degradable compounds and chemotherapeutics are being developed. Currently, a small number of anticancer nanopharmaceuticals mainly carrying chemotherapeutics and not aimed at ECM modulation are FDA/EMEA approved and marketed.

Clinical trials of nanopharmaceuticals targeting the ECM are awaited as preclinical studies of mouse tumour xenografts have demonstrated improved tissue penetration and inhibition of tumour growth. Concerns regarding an increased risk of dissemination and accumulation of nanoparticles in normal tissues that could potentially cause unwanted side effects are currently questioning the long-term safety.

Improving the binding efficiency of target ligands will lead to increased selectivity and precise drug release. ECM-modulatory regimens that increase local drug release and improve the penetration of cytotoxic immune cells may advantageously be combined with targeted immune therapy; however, we need to explore further the differences in the ECM composition between immunogenic hot and cold tumours.

As presented, specific combinations of biophysical and biochemical signals induce cancer cell dissemination and migration, emphasising the importance of current research to map the complex biological structures of the ECM and the TME. Furthering our understanding of the dynamics within the TME between the ECM and tumour cells in space and time accelerates the search for novel targeted approaches. The application of high-tech and high-resolution imaging techniques will elucidate the role of ECM protein organisation, provide information at subcellular levels regarding the complex single-cell phenotypes and their spatial context, and aid in our efforts to quantify tissue structural changes during disease progression.

Research into the ECM is currently aided by an increase in omics being developed. Spatial transcriptomics and proteomics characterise expression down to individual cells or even subcellular locations. For the ECM, new omics, including the matrisome and adhesome (molecules involved in cell-to-ECM adhesion), allow researchers to quickly identify potential targets and guide new experimental designs.

It is our opinion that the progress in oncology, cell biology, and material sciences will pave the way for an interdisciplinary integration of ECM characteristics in personalised treatment options.

## 12. Conclusions

The composition and function of ECM change dramatically during the development of solid cancers, supporting both the growth and dissemination of cancer cells. Research into the biochemical and biophysical properties during all stages of carcinogenesis is essential to furthering our understanding of the disease and for the integration of ECM profiling into future individualised treatment strategies. This review focuses on the recent advances in ECM research and potential treatment targets.

## Figures and Tables

**Table 1:** Growth factors and their association with ECM proteins.

GF	ECM Protein	Growth Factor Functions
TGFβ	Type IV collagen Heparin/HS Fibronectin Fibrin/fibrinogen Betaglycan Decorin	Modulates cell growth and differentiation. Stimulates the synthesis of collagen, fibronectin and other ECM components, including HA, TSP, and tenascin. Increases production of protease inhibitors. Reduces the synthesis and secretion of proteases.
HGF	Type I, III, IV, V, and VI collagen Heparin/HS Fibronectin Fibrin/fibrinogen	Stimulates matrix remodelling and epithelial regeneration. Inhibits fibrosis.
IGF	IGF-binding protein	Stimulates cell mitogenesis, differentiation, and survival. Amplifies activity through the engagement of integrins or ECM glycosaminoglycans, heparin-binding domains, affecting cell adhesion and migration.
PDGF	SPARC Heparin/HS Fibronectin Fibrin/fibrinogen	Regulates angiogenesis. Attracts fibroblasts and monocytes and accelerates granulation tissue formation and ECM deposition.
VEGF	Collagen Heparin/HS Fibronectin Fibrin/fibrinogen	Controls blood vessel formation and growth. Binds to fibronectin to synergistically promote endothelial cell proliferation.
EGF	Collagen Fibronectin	Stimulates epithelial cell proliferation. Regulates a subset of G1 cell-cycle events. Elevates levels of EGFR.
FGF	Heparin/HS Fibronectin Fibrin/fibrinogen	Induces fibroblast proliferation and angiogenesis. Oligomerisation prolongs activity protection from proteolysis and endocytosis.

EGF: epidermal growth factor; EGFR: EGF-receptor; FGF: fibroblast growth factor; HA: hyaluronic acid; HGF: hepatocyte growth factor; HS: heparin sulphate; IGF: insulin-like growth factor; PDGF: platelet-derived growth factor; SPARC: secreted protein acidic and rich in cysteine, TGFβ: transforming growth factor beta; VEGF: vascular endothelial growth factor.

**Table 2:** A non-exhaustive table summarising the expression levels of some of the main signalling pathways in various solid cancers that are affected by ECM dysregulation, such as aberrant collagen deposition, increased HA expression, and abnormal expression of laminin and fibronectin. It is important to note that the expression levels of signalling pathways may vary. They are not mutually exclusive, and as shown, they often overlap and interact with each other. Signalling pathways are also affected by the accumulation of genetic mutations and disease progression and may change their oncogenic drive. With progression, changes in the composition and organisation of the ECM may affect additional pathways. For example, in early-stage breast cancer, changes in collagen I and IV deposition can activate the PI3K/AKT and MAPK/ERK pathways, whereas in advanced stages, the deposition of different types of collagens, such as collagen VI, can activate the TGF-β pathway. Similarly, in lung cancer, the expression of hyaluronan is increased in advanced stages, leading to dysregulation of the Hippo pathway, which promotes tumour growth and metastasis. CRC: colorectal cancer, EMT: epithelial-mesenchymal transition, EGFR: epidermal growth factor receptor, ERK: extracellular signal-regulated protein kinase, FAK: focal adhesion kinase, HA: hyaluronic acid, HCC: hepatocellular carcinoma, MAP/ERK: microtubule-associated protein kinase; MAPK: mitogen-activated protein kinase, NSCLC: non-small cell lung cancer, PDAC: pancreatic ductal adenocarcinoma, PI3K/Akt: phosphoinositide 3 kinase/protein kinase B (originally Ak strain transforming kinase), Ras/Raf: rat sarcoma virus protein/rapidly accelerated fibrosarcoma protein kinase, RhoA/ROCK: Rho GTPase A/serine-threonine protein kinase, SMAD: Suppressor of Mothers against Decapentaplegic, Src: non-receptor cytoplasmic tyrosine kinase, YAP: Yes-associated protein, TGF-β: transforming growth factor. Wnt: wingless-related integration site.

Signalling Pathways Affected by ECM Dysregulation	Breast Cancer	NSCLC	CRC	PDAC	Prostate Cancer	Ovarian Cancer	HCC	Glioblastoma	Malignant Melanoma
EMT	+		+	+		+	+	+	
EGFR			+				+	+	
FAK	+			+		+			
FAK/Src			+		+	+		+	+
HA		+							
Hedgehog	+			+	+	+		+	
Hippo		+						+	+
Hippo/YAP			+						
Integrin	+	+	+	+	+	+	+	+	
MAPK/ERK	+		+				+		+
Notch				+	+			+	
PI3K/AKT	+	+	+	+	+	+	+	+	+
RAS/RAF/MAPK/ERK		+						+	
RhoA/ROCK					+				
Rho GTPase	+								
TGF-β	+				+	+		+	+
TGF-β/Smad			+			+	+		
Wnt/β-catenin	+	+	+	+	+	+	+	+	

**Table 3:** Recently completed clinical trials targeting ECM (not including already approved and marketed drugs).

Trial No and Phase	Cancer Type	Drug	Target	Mechanism of Action	Preliminary Results and Adverse Events
NCT00431561; Phase 2b	Recurrent and refractory glioma	Trabedersen (AP 12009)	TGFβ2	Antisense oligodeoxynucleotide specifically inhibits TGF-beta2 and suppresses key mechanisms of tumour development, specifically immunosuppression, metastasis, angiogenesis, and proliferation.	19/89 pts had CR or PR following robust lesion size reduction (med. time for 90% reduction of baseline tumour volume = 11.7 mo, 4.9–57.7 mo). 7 pts had an SD for ≥6 mo. For the group of 26 AA/GBM patients with favourable responses, the median PFS: 1109 days and OS: 1280 days (significantly better than seen in the group including non-responders (*n* = 89; *p* < 0.00001) (https://doi.org/10.3390/cancers11121892)
NCT00761280; Phase 3	Recurrent or refractory AA and secondary glioblastoma	Trabedersen + Temozolomide + Carmustine + Lomustine			Terminated due to inability to recruit the projected patient number.
NCT01401062; Phase 2	m-Breast cancer	Fresolimumab + Focal irradiation	TGFβ	Human IgG4-κ monoclonal antibody neutralises all TGFβ isoforms (i.e., β1, β2, and β3) with half-life ranging from 21–30 days.	7 grade 3/4 AE in 5/11 pts (1 mg/kg arm) and in 2/12 pts (10 mg/kg arm), respectively. SD = 3. At 12 months follow-up, 20/23 pts deceased. Patients receiving the 10 mg/kg had a significantly higher OS than those receiving 1 mg/kg fresolimumab (HR: 2.73 with 95% CI: 1.02, 7.30; *p* = 0.039). (https://doi.org/10.1158/1078-0432.CCR-17-3322)
NCT01112293; Phase 2	Relapsed malignant pleural mesothelioma	Fresolimumab (GC1008)			SD: 3/13 pts; serum from 5 patients showed increased levels of antibodies against MPM tumour lysates. Had increased OS (15 vs. 7.5 mo, *p* < 0.03) (https://doi.org/10.4161/onci.26218)
NCT01246986; Phase 2	HCC	Galunisertib + Sofarenib + Ramucirumab	TGFβ-R1	Galunisertib (LY2157299) acts as a small-molecule selective inhibitor of the TGF-β receptor type I, which is a serine/threonine kinase.	Median time-to-tumour progression was 4.1 mo for 150 mg Galunisertib cohort; OS: 18.8 mo; PR: 2 pts; SD: 21; and progressive disease: 13. TGF-β1 responders showed better OS compared to non-responders (22.8 vs. 12.0 months, *p* = 0.038). (https://doi.org/10.14309/ctg.0000000000000056)
NCT01373164; Phase 1, 2	m-Neoplasms, pancreatic cancer	Galunisertib + Gemcitabine/ Placebo + Gemcitabine			OS: 10.9 vs. 7.2 mos (GG vs. GP) in the subgroup with baseline TGFβ1 levels ≤ 4224 pg/mL (*n* = 117). PFS: 3.65 vs. 2.79 mos (*p* = 0.215). ORR: 8.7 vs. 1.9 (*p* = 0.116). Grade 3/4 TR-AE (GG vs GP) were anaemia (7.8% vs. 13.5%), neutropenia (32.0% vs. 26.9%) and thrombocytopenia (7.8% vs. 9.6%).
NCT02149108; Phase 3	Refractory m-CRC	Nintedanib (BIBF1120)/ Placebo	RTK	Oral small-molecule inhibitors of RTK, including FGFR-1 to 3, PDGFR-α and β, and VEGFR-1 to 3. It inhibits the release of proinflammatory and profibrotic mediators, migration and differentiation of fibrocytes and fibroblasts, and deposition of ECM.	OS 6.4 mo vs. 6.0 mo with placebo; HR 1.01; 95% CI 0.86–1.19; *p* = 0.8659. PFS 1.5 mo vs. 1.4 mo; HR 0.58; 95% CI 0.49–0.69; *p* < 0.0001). No CR or PR. AEs occurred in 97% of the treatment group (*n* = 384) and 93% of the placebo group (381). The most frequent grade ≥ 3 AEs were liver-related AEs (nintedanib 16%; placebo 8%) and fatigue (nintedanib 9%; placebo 6%). (https://doi.org/10.1093/annonc/mdy241)
NCT01015118; Phase 3	Ovarian and peritoneal neoplasms	Nintedanib/Placebo + Carboplatin + Paclitaxel			53% of nintedanib grp (486/911) had disease progression or death compared with 58% of placebo grp (266). PFS: 17.2 mo vs. 16.6 mo, HR 0.84; 95% CI 0.72–0.98; *p* = 0.024. The most common AE were diarrhoea, neutropenia, anaemia, and thrombocytopenia. SAE in 376/902 pts in nintedanib grp and 155/450 pts in placebo grp. 29 pts in the nintedanib group had SAE compared with 16 in the placebo group. TR-AE-related death in 3 vs. 1 pts in treatment vs the placebo group.
NCT01195415; Phase 2	m-Pancreatic cancer	Vismodegib/Placebo + Gemcitabine	SMO	Vismodegib selectively binds to and inhibits the transmembrane G protein-coupled receptor protein, SMO, to inhibit the Hedgehog signalling pathway and reduce desmoplasia.	75% pts had elevated SHH expression pretreatment. Post-treatment, GLI1 and PTCH1 decreased in 95.6% and 82.6% of 23 pts, fibrosis decreased in 45.4% of 22 and Ki-67 in 52.9% of 17 evaluable pts. PFS and OS for all pts was 2.8 and 5.3 mos. DCR: 65.2%. Grade > 3 AE seen in 56% pts. (*n* = 23) (https://doi.org/10.1158/1078-0432.CCR-14-1269)
NCT02667574; Phase 2	laBCC	Vismodegib + Surgery			44/55 patients had a procedure after vismodegib treatment (80.0%, 95% CI [67 to 90]). CR: 27/44. The main AEs were dysgeusia, muscle spasms, alopecia, fatigue, and weight loss (20% pts with grade ≥ 3). Vismodegib helps with downstaging before surgery for laBCC. (https://doi.org/10.1200/JCO.2018.36.15_suppl.9509)
NCT01130142, Phase 1, 2	m-Pancreatic cancer	Patidegib (IPI-926) + Gemcitabine	SMO	Small-molecule, semi-synthetic cyclopamine analogue that inhibits SMO	Preliminary results: 3/9 radiographic PR with post-baseline scans. No Grade 4/5 AE and no TR-AEs. The most common TR-AEs: fatigue (40% total, 0% Grade 3), nausea (40%, 0%), ALT increased (13%, 7%), AST increased (13%, 7%), anaemia (13%, 0%), and vomiting (13%, 0%). The study was terminated early due to a lack of benefits.
NCT01479465; Phase 2a	m-CRC	A. Simtuzumab 700 mg + FOLFIRIB. Simtuzumab 200 mg + FOLFIRIC. Placebo + FOLFIRI	LOXL2	A humanised IgG4 monoclonal antibody against LOXL2 inhibits its enzymatic activity and ECM remodelling required for tumour progression.	PFS: A. 5.5 mo, B. 5.4 mo, and C. 5.8 mo. OS: 11.4 mo (1.23 [0.80, 1.91]; *p* = 0.25), 10.5 mo (1.50 [0.98, 2.30]; *p* = 0.06), and 16.3 mo. ORR was 11.9%, 5.9%, and 10%. Simtuzumab was well tolerated; however, clinical outcomes did not improve. (https://doi.org/10.1634/theoncologist.2016-0479)
NCT00195091, Phase 2	Breast Cancer, TNBC	Tetrathiomolybdate	LOX	TM is a copper chelator that targets the catalytic activity of LOX by binding to copper and depleting it.	Disease progression was seen in 14/74, and 17 died. EFS: 71.4% and OS: 64.7% for all patients. Cancer-specific OS: 79.9%. TNBC: EFS: 71.7%, and OS: 74.2%. non-TNBC: EFS: 71.2% and OS: 64.6%
NCT01839487, Phase 2	m-PDAC	PEGPH20 plus nab-paclitaxel/gemcitabine (PAG) or nab-paclitaxel/gemcitabine (AG)	Hyaluronic acid	PEGPH20 degrades tumour-associated HA and increases the efficacy of chemo- and immuno-therapeutic agents.	PFS significantly improved with PAG (HR, 0.73; 95% CI, 0.53–1.00; *p* = 0.049) and for patients with HA-high tumours. ORR: 45% vs. 31% (PAG vs. AG). OS: 11.5 vs. 8.5 mos (HR, 0.96; 95% CI, 0.57–1.61). The most common grade 3/4 TR-AE with significant differences between arms (PAG vs. AG): muscle spasms (13% vs. 1%), neutropenia (29% vs. 18%), and myalgia (5% vs. 0%) (https://doi.org/10.1200/JCO.2017.74.9564)

AA: anaplastic astrocytoma; AE: adverse events; AMPK: AMP-activated protein kinase; ALT: alanine aminotransferase; AST: aspartate aminotransferase; BTC: biliary tract cancer; laBCC: locally advanced basal cell carcinoma; CI: confidence interval; CR: complete response; CRC: colorectal cancer; DCR: disease control rate; FGFR: fibroblast growth factor receptor; GBM: glioblastoma multiforme; GG: gemcitabine plus galunisertib; GP: gemcitabine plus placebo; GIST: gastrointestinal solid tumors; HIF: hypoxia inducing factor; HCC: hepatocellular carcinoma; HNSCC: head and neck squamous cell carcinoma; HR: hazard ratio; LOXL2: lysyl oxide like 2; ICI: immune checkpoint inhibitor; IGFR: insulin-like growth factor receptor; IRTK: insulin receptor tyrosine kinase LUSC: squamous cell lung carcinoma; m: metastatic; MET: hepatocyte growth factor receptor; mTOR: mammalian target of rapamycin; mTORC1/2: mTOR complex 1/2; NFE2L2/NRF2: Nuclear factor erythroid 2-related factor 2; NSCLC: non-small cell lung cancer; ORR: overall response rate; OS: overall survival; PD1: programmed cell death protein 1; PDL1: programmed death ligand 1; PDGFR: platelet-derived growth factor receptor; PR: partial response; RCC: renal cell carcinoma; RTK: receptor tyrosine kinase; SD: stable disease; SMO: smoothened TGFβ transforming growth factor-beta; TNBC: triple negative breast cancer; TRKB: tropomyosin-related kinase B; TR-AE: treatment related AE; VEGFR: vascular endothelial growth factor receptor.

## Data Availability

All data presented in this manuscript are accessible in the National Library of Medicine at www.pubmed.ncbi.nlm.nih.gov (accessed on 1 February 2023).
